# The study on the molecular characteristics and variation patterns of the recombinant Muscovy duck parvovirus strain GD-23

**DOI:** 10.1080/21505594.2025.2530666

**Published:** 2025-07-15

**Authors:** Mingtian Mao, Jiake Li, Caiqi Wang, Mian Wu, ChengGuang Lu, Yudong Zhu, HuiHui Li, Bing Li, Meixi Lu, Yi Tang

**Affiliations:** a College of Animal Science and Technology, Shandong Agricultural University, Tai’an, Shandong, China; b Shandong Provincial Key Laboratory of Animal Biotechnology and Disease Control and Prevention, Shandong Agricultural University, Tai’an, Shandong, China; c Shandong Provincial Engineering Technology Research Center of Animal Disease Control and Prevention, Shandong Agricultural University, Tai’an, Shandong, China; d Institute of Animal Science, Chinese Academy of Agricultural Sciences, Beijing, China

**Keywords:** Recombinant Muscovy duck parvovirus (rMDPV), isolation, pathogenicity analysis, recombination, AlphaFold 3, amino acid mutations

## Abstract

As the virus continues to spread, the frequency of contact between different parvovirus strains has increased. In recent years, recombinant Muscovy duck parvovirus (rMDPV) has been widely prevalent in China. In 2023, a strain of rMDPV, designated GD-23, was isolated from Guangdong. After isolation and culture, the entire genome of GD-23 was sequenced. A phylogenetic tree was constructed based on the genomic characteristics of GD-23, and recombination events were analysed. The analysis revealed that the virus underwent two recombination events between different parent strains in the P9 promoter region and part of the VP3 region, with highly consistent start and end breakpoints among rMDPV strains. Using AlphaFold3, the VP3 protein conformation of waterfowl parvoviruses (WPVs) was simulated, and potential receptor interaction regions were predicted based on the structural similarities of capsid proteins among viruses of the same genus. Notably, the rMDPV-GD-23 strain exhibited two identical amino acid mutations at distinct sites, which reduced the overall phosphorylation of the viral capsid. Artificial infection experiments in waterfowl demonstrated that the rMDPV-GD-23 strain was pathogenic to multiple waterfowl hosts. This study provides the first evidence that rMDPV possesses cross-host infection capability, a finding that will aid in identifying key amino acid sites on the capsid surface of WPVs.

## Introduction

Parvoviruses have persisted on Earth for tens of millions of years, infecting a broad range of animal hosts across diverse taxa [1,2]. Among these, goose parvovirus (GPV) and Muscovy duck parvovirus (MDPV) specifically target waterfowl species and have recently been reclassified under the *Dependoparvovirus genus* (*Parvoviridae family*) based on updated phylogenetic criteria [3,4]. Both viruses possess a 5.1 kb linear single-stranded DNA genome flanked by inverted terminal repeats (ITRs), with GPV and MDPV ITRs measuring 444 and 455 nt in length, respectively [5]. These ITRs form palindromic secondary structures that serve as replication origins, enabling viral genome amplification via host DNA polymerase [6]. The genomic architecture of GPV and MDPV contains two major open reading frames (ORFs): the left ORF encodes the non-structural proteins NS1 and NS2, whereas the right ORF produces structural proteins VP1, VP2, and VP3 through the alternative splicing of a shared transcript. These structural proteins, which share identical C-terminal domains but differ in their N-terminal regions, assemble into icosahedral capsids at a stoichiometric ratio of 1:1:8 (VP1:VP2), forming a mature virion structure [7,8].

Over the past two decades, recombinant MDPV has emerged as a novel pathogen in waterfowl populations across China, particularly affecting members of the order *Anseriformes* [9]. This emergence stems from multiple factors, the chief of which are the overlapping ecological niches and genetic compatibility between MDPV and GPV. Specifically, MDPV is a causative agent of motor dysfunction and enteric disease with high morbidity in ducklings under three weeks of age [10]. Shares partial host susceptibility to GPV, which exhibits cross-species tropism in Muscovy ducks [11]. The co-circulation of these viruses facilitates homologous recombination events during dual infections, generating novel chimeric strains with enhanced replication fitness and expanded host range [9]. Clinically, rMDPV infection manifests as a bimodal pathology in ≤ 30-day-old Muscovy ducks, with mortality rates reaching 60%. A minority of cases (5–15%) present with beak dysplasia and alopecia syndrome, whereas the predominant phenotype (85–95%) resembles GPV-induced fibrinous enteritis. [12, 13]. This pathological overlap has led to the colloquial designation of some rMDPV variants as “Muscovy duck-derived goose parvovirus” (MD-GPV) in Chinese veterinary contexts.

WPVs and adeno-associated viruses (AAV) share taxonomic classification within the *Dependoparvovirus genus* (*Parvoviridae family*), with emerging evidence suggesting structural and functional parallels between these viral groups. Recent studies have identified AAVR as the primary cellular receptor for AAV, a discovery driven by its utility in gene therapy vector design [14]. Comparative genomic analyses reveal ~ 60% amino acid sequence homology between the VP (viral protein) genes of WPVs and AAV, accompanied by conserved *T* = 1 icosahedral symmetry in their capsid architectures [1,15]. These phylogenetic and structural convergences suggest conserved surface topographies that may mediate analogous host cell interactions through the stereochemical complementarity of capsid epitopes.

Our current understanding of the biological characteristics and whole-genome analysis of rMDPV in China is still limited, with research on the cross-host infection mechanisms of this recombinant pathogen being scarce. We conducted whole-genome sequencing of a 2023 rMDPV strain isolated in our laboratory. By combining recombination analysis with protein conformation modelling, we mapped key amino acid sites on the capsid surface of WPVs using simulated rMDPV-AAVR interaction regions as structural benchmarks. The potential for cross-species infection was preliminarily assessed using animal infection experiments.

## Materials and methods

### Sample collection

The liver tissue samples used in this study were obtained from a Muscovy duck farm in Guangdong Province. In July 2023, the Muscovy ducks on this farm experienced a mass mortality event, with a mortality rate exceeding 90% among 11-day-old ducklings. Postmortem examination revealed obvious pathological features of intestinal thrombosis in the intestines. Samples were collected from dead ducklings with typical post-mortem symptoms. Liver tissues were immediately placed into cryotubes containing RNA protectant and transported to the laboratory via dry ice cold chain for subsequent research.

### Pathogen proliferation

The tissues were homogenized and resuspended in a complete serum-free medium. The viral suspension was centrifuged at 8,000 × g for 10 min, followed by filtration of the supernatant. A 1 mL aliquot of the filtered viral suspension was inoculated onto DEF cells (Duck Embryo Fibroblasts, ATCC CCL-141), followed by incubation at 37°C for 5 days. The cell culture supernatant was collected and subjected to three freeze-thaw cycles at −80°C, followed by centrifugation to remove cellular debris. The virus suspension was blind-passaged for three generations under identical conditions, with the final viral preparation stored at −80°C for future use.

### Viral growth curve

The multi-step growth kinetics of the isolates were assessed in DEF cells using the median tissue culture infectious dose (TCID50) [16]. Cell culture supernatants were harvested at 12, 24, 36, and 48 h post-infection (hpi). Following three freeze-thaw cycles, the samples were stored at −80°C. DEF cells were seeded in 96-well plates at a density of 10^5^ cells with 100 µL of complete medium per well and incubated at 37°C under a 5% CO_2_ atmosphere for 24 h. Following the removal of the culture medium, 100 µL of 10-fold serial dilutions of the viral inoculum collected at different time points were added to the wells. Cytopathic effects (CPE) were assessed at 48 hpi, and viral titres were calculated using the Reed-Muench method.

### DNA extraction and sequence amplification

Viral genomic DNA was isolated from 250 μL of the viral suspension using phenol-chloroform-isoamyl alcohol (25:24:1, v/v). Purified DNA was analysed using polymerase chain reaction (PCR) amplification. Specific primers targeting viral genome sequences were designed (sequences are provided in Supplementary Table S1). The thermal cycling protocol consisted of an initial denaturation at 95°C for 5 min, followed by 30 cycles of denaturation at 95°C for 30 s, annealing at 55°C for 1 min, and extension at 72°C for 2 min, with a final extension at 72°C for 10 min. The PCR products were analysed using 1% agarose gel electrophoresis.

### Whole genome sequencing and analysis

PCR amplicons were purified using a gel extraction kit, followed by ligation into the pMD18-T vector (Takara, Dalian, China). The recombinant plasmids were transformed into chemically competent Escherichia coli SURE cells (Stratagene, La Jolla, CA, USA). Three independent clones were submitted to BGI Tech (Shenzhen, China) for bidirectional sequencing. Multiple sequence alignment and homology analysis were conducted with Clustal W within the DNASTAR Lasergene 5.0 suite (Madison, WI, USA). Phylogenetic reconstruction was performed using maximum likelihood algorithms implemented in MEGA 6.0 [17]. Reference parvovirus strains are detailed in Supplementary Table S2.

### Recombination analysis

Potential recombination signals in the viral genome were screened using the whole-genome consensus sequence analysed with RDP4. The analysis incorporated seven detection algorithms: RDP, GENECONV, BootScan, MaxChi, Chimaera, SiScan, and PhylPro. Putative recombination events were considered significant only when identified by ≥ 5 algorithms with concordant breakpoints and statistical support (*p* < 1.0 × 10^^−14^). Topological validation of recombination breakpoints was performed through bootscanning analysis using the SimPlot software. Phylogenetic verification of recombinant regions was conducted by maximum-likelihood tree construction using genome segments flanking the identified breakpoints [17].

### Simulation of protein structure

The AlphaFold 3 model can predict the joint structures of complexes with higher accuracy than previous generations [18]. Leveraging the AlphaFold 3 online platform (AlphaFold Server (google.com)) for amino acid three-dimensional structure simulation, we comprehensively modelled the VP3 protein structures for AAV2, AAVR-PKD2, classical waterfowl parvovirus (GPV-Yan-2/MDPV-P1/NGPV-KC3S3), and rMDPV-GD23. The modelling results were processed and analysed using the PyMOL software [19]. The reasonableness of the modelling results was assessed using the SAVES v6.0 online Ramachandran Plot generation platform (SAVESv6.0 - Structure Validation Server (ucla.edu)), and the evaluation results are listed in Table S3.

### Protein docking simulation

Based on the parvovirus capsid arrangement properties [14], protein – protein docking simulations between AAVR-PKD2 and rMDPV-VP3 were performed using AlphaFold 3. Docking reliability was evaluated using the interface prediction TM-score (ipTM) and predicted template modelling score (pTM), with a combined threshold of ipTM + pTM > 0.75 established as the credibility cut-off [20]. Interaction regions were analysed using PyMOL software and visualized on individual rMDPV-VP3 capsomers.

### Antibody neutralization assay

To investigate the potential role of AAVR as a receptor for rMDPV, we initially constructed a prokaryotic expression system by cloning the AAVR gene into pET-30a vector. The full-length 945bp PKD2 domain sequence was amplified using specific primers (PKD2-F:TACAGTTATGCTACCCCTACCCCCC; PKD2-R: AGGAGGCTTATTGTTTTCAGGTTGC), corresponding to GenBank accession number NM_024874.5. The recombinant AAVR protein was purified using density gradient centrifugation and subsequently used to subcutaneously immunize BALB/c mice for polyclonal antibody production. Target IgG antibodies were isolated from the antiserum using Protein G affinity chromatography.

Three experimental groups were established: (1) negative control (PBS), (2) positive control (AAVR antibody pre-incubated with AAVR protein at 37°C for 1 h), and (3) test group (AAVR antibody alone). All groups were pre-incubated with DEF cells at 37°C for 1 h prior to infection with rMDPV particles at an MOI of 1. After 72 h post-infection, viral nucleocapsid protein expression was quantified by indirect immunofluorescence assay (IFA) using a laboratory-prepared rMDPV-VP3 mouse polyclonal antibody as the primary antibody and FITC-conjugated goat anti-mouse IgG (H+L) as the secondary antibody. The blocking efficacy of the AAVR antibody was evaluated through microscopic examination of cytopathic effects (CPE) and fluorescence intensity measurements.

### Waterfowl infection experiment

Pathogen: rMDPV-GD23 (GenBank accession number: PP763298) was isolated and identified in this study.

Animals: One-day-old healthy Cherry Valley ducks (*n* = 63), Muscovy ducks (*n* = 63), and geese (*n* = 63) were purchased from a commercial farm and housed in cages. The parents of the test animals were vaccinated against GPV within one week of hatching. On the day of arrival, three animals were euthanized, and serum samples were tested for maternal neutralizing antibodies against GPV using a serum neutralization test. Specific qPCR for the virus was performed on meconium, intestinal, and liver samples to confirm that the test animals were free of parvovirus infection.

To evaluate the characterization of rMDPV infection in different waterfowl, geese, Muscovy ducks, and Cherry Valley ducks were randomly divided into two groups, with 30 birds per species in each group. The experimental group (30 geese, 30 Muscovy ducks, and 30 Cherry Valley ducks; total, 90 birds) was orally inoculated with rMDPV-GD23 (1,000 TCID_50_) to simulate a natural infection. In contrast, the control group (30 geese, 30 Muscovy ducks, and 30 Cherry Valley ducks; total = 90 birds) received an equivalent oral dose of sterile PBS. The animals were grouped and maintained in completely identical environments, and monitoring was conducted over a continuous period of 28 days. Daily assessments focused on the individual size, mortality counts, and clinical symptoms. All animals from both the experimental and control groups were included in the final analyses. At the end of the study, all surviving animals were euthanized.

On days 7, 14, 21, and 28 post-infection, three experimental animals from each group were randomly selected for weight measurements, euthanasia, and necropsy. During necropsy, fresh tissue samples were collected from each animal to measure the viral load.

### Statistical analysis

Statistical significance between the experimental groups was determined using a t-test. Viral loads were statistically analysed using the t-test with GraphPad Prism version 8.0 (GraphPad Software Inc.). Statistical significance was set at *p* < 0.05 and *p* < 0.01.

## Results

### Isolation and identification of rMDPV

**Figure 1.**
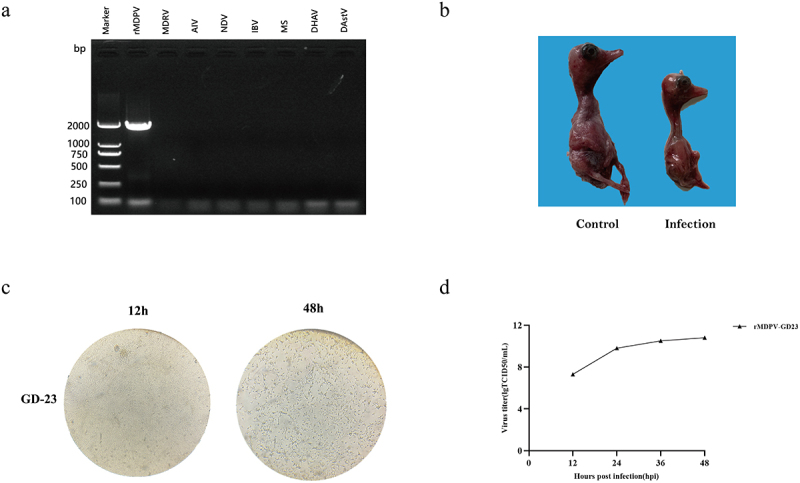
Characteristics of rMDPV-GD-23 strain in DEF cell culture. (a) Detection of various waterfowl-derived viruses via PCR following infection of DEF cells with the viral isolate (b) Pathological manifestations observed in 10-day-old Cherry Valley duck embryos post-infection with rMDPV-GD23 (c) Cytopathic effects of rMDPV-GD23 on DEF cells at 48 hours post-infection (hpi) (d) Growth kinetics of rMDPV-GD23 in DEF cells assessed at 12, 24, 36, and 48 hpi.

In recent years, Muscovy duck mortality caused by rMDPV has markedly increased in southern China. Viral suspensions from Guangdong samples were inoculated into DEF cells, with pronounced CPE observed from the third passage onward. These effects were characterized by widened intercellular spaces and reduced cell adhesion. The isolated virus, designated GD-23, tested positive for rMDPV by PCR but was negative for common waterfowl pathogens, including Muscovy duck reovirus, avian influenza virus, Newcastle disease virus, infectious bronchitis virus, Mycoplasma synoviae, duck hepatitis A virus, and duck astrovirus (Figure 1a). Following five serial passages, GD-23 was aseptically inoculated into 10-day-old Cherry Valley duck embryos via the allantoic cavity, resulting in significant growth inhibition and haemorrhagic manifestations in the infected embryos (Figure 1b). Inoculation of GD-23 into DEF cells induced prominent CPE within 48 h (Figure 1c). Multi-step growth curves generated from TCID_50_ values at 12, 24, 36, and 48 hours post-infection (hpi showed viral replication in Cherry Valley duck embryonic fibroblasts, reaching peak titers at 48 hpi (Figure 1d). These results demonstrate that the rMDPV-GD-23 strain exhibits high adaptability to the Cherry Valley duck cell culture system.

### Phylogenetic analyses

**Figure 2.**
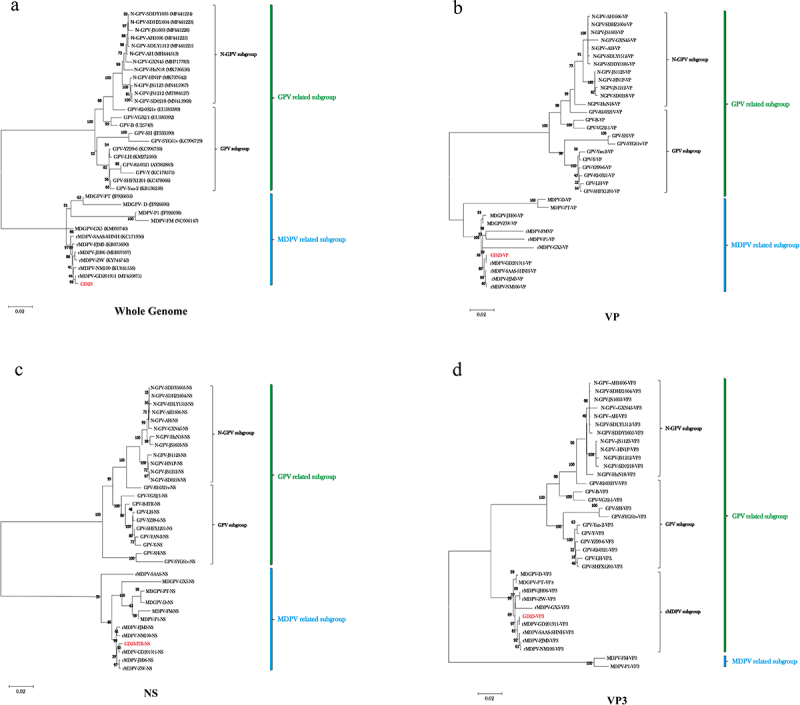
Phylogenetic tree construction based on WPV gene sequences from GenBank a phylogenetic tree was constructed using the maximum likelihood method. Bootstrap majority consensus values from 1,000 replicates are presented as percentages at each branch point, with a scale bar indicating the number of nucleotide substitutions per site. The red font denotes rMDPV-GD23 identified from the infected Muscovy duck in this study.

Phylogenetic analysis elucidated the evolutionary relationships between the GD-23 wild strain and the reference WPV strains. Phylogenetic trees were constructed using the maximum likelihood method based on the whole genome, NS, VP, and VP3 sequences (Figure 2). Current findings indicate that WPV evolution clusters into two subgroups (GPV and MDPV), suggesting no emergence of strains with distinct evolutionary patterns in recent years. Analyses of whole-genome, NS, and VP sequences demonstrated that GPV and the 2014-emerged NGPV cluster within the GPV subgroup, whereas MDPV follows an independent evolutionary trajectory (Figure 2a–c). Notably, the VP3-based phylogenetic tree revealed substantial discrepancies compared to other analyses (Figure 2d). Within the MDPV subgroup, rMDPV clustered with GPV strains, leaving only two classical MDPV strains (MDPV-FM and MDPV-PT) in the original subgroup. Further analysis identified three evolutionary trajectories in the rMDPV branch, with GD-23 diverging from both MD-GPV strains (MD-GPV-D and MD-GPV-PT) and earlier classical rMDPV (rMDPV-ZW). These results suggest novel evolutionary patterns of rMDPV in recent years.

### Recombination analysis

**Figure 3.**
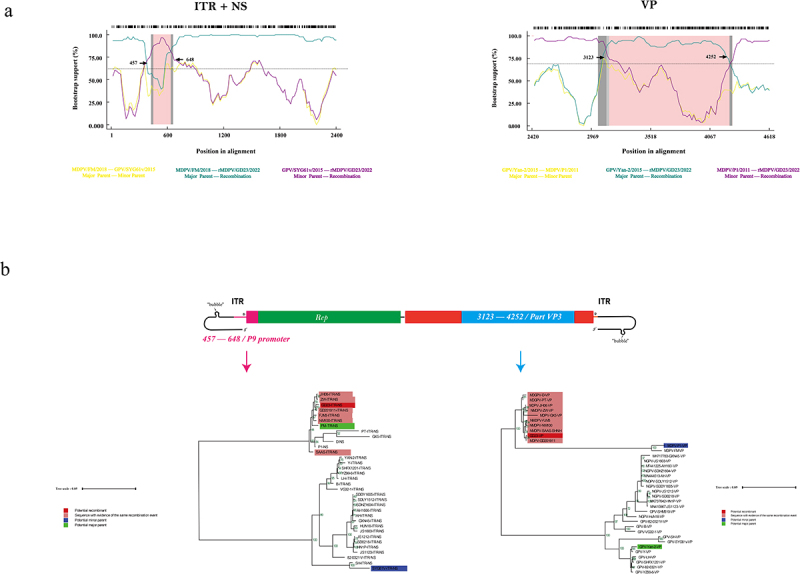
Recombination analysis of the GD-23 strain of recombinant Muscovy duck parvovirus. (a) results from RDP4 showing possible recombination events in GD-23. (b) Maximum likelihood trees were constructed based on the recombination region.

To investigate potential recombination events within the gene fragments of the GD-23 strain, the VP gene region (used for phylogenetic analysis) and the P9 promoter region (spanning ITR and NS segments) were analysed using RDP4 [13]. Two recombination events were identified in the GD-23 genome, each involving distinct primary and secondary parental strains (Figure 3a). The first recombination event (457–648 nt) was observed between the classical MDPV strain FM (primary parent) and GPV attenuated vaccine strain SYG61v (secondary parent). The second event (3123–4252 nt) involved the classical MDPV strain P1 (primary parent) and classical GPV strain Yan-2 (secondary parent). These findings suggest that viral recombination is a complex and dynamic process characterized by frequent inter-strain recombination within high-frequency recombination regions during viral epidemics. The recombination events were further validated using SimPlot software, which yielded results consistent with those obtained using RDP4 analysis.

Maximum likelihood phylogenetic trees were reconstructed for both recombination regions using RDP4 (Figure 3b). All rMDPV strains showed conserved recombination patterns in the P9 and VP genomic regions. However, MD-GPV representative strains (MD-GPV-D and MD-GPV-PT) showed no recombination events in the P9 promoter region compared to other rMDPVs. Notably, parental strains for these events were absent from the analysed reference strains. These findings suggest that MD-GPV strains may possess unique biological characteristics that distinguish them from other rMDPV lineages.

Research indicates that the formation mechanism of the GD-23 strain involves genomic recombination events between the classical MDPV strain, attenuated GPV vaccine strain, and classical GPV strain in the P9 promoter region and VP nucleocapsid protein-coding region. This characteristic aligns with the typical recombination pattern observed in rMDPV strains. Notably, the persistent recombination phenomena observed in MDPV reveal an important viral evolutionary pattern: large-scale genomic recombination between closely related viral strains is a key mechanism driving the emergence of new viral strains during the evolution of DNA viruses.

### Protein docking analysis

**Figure 4.**
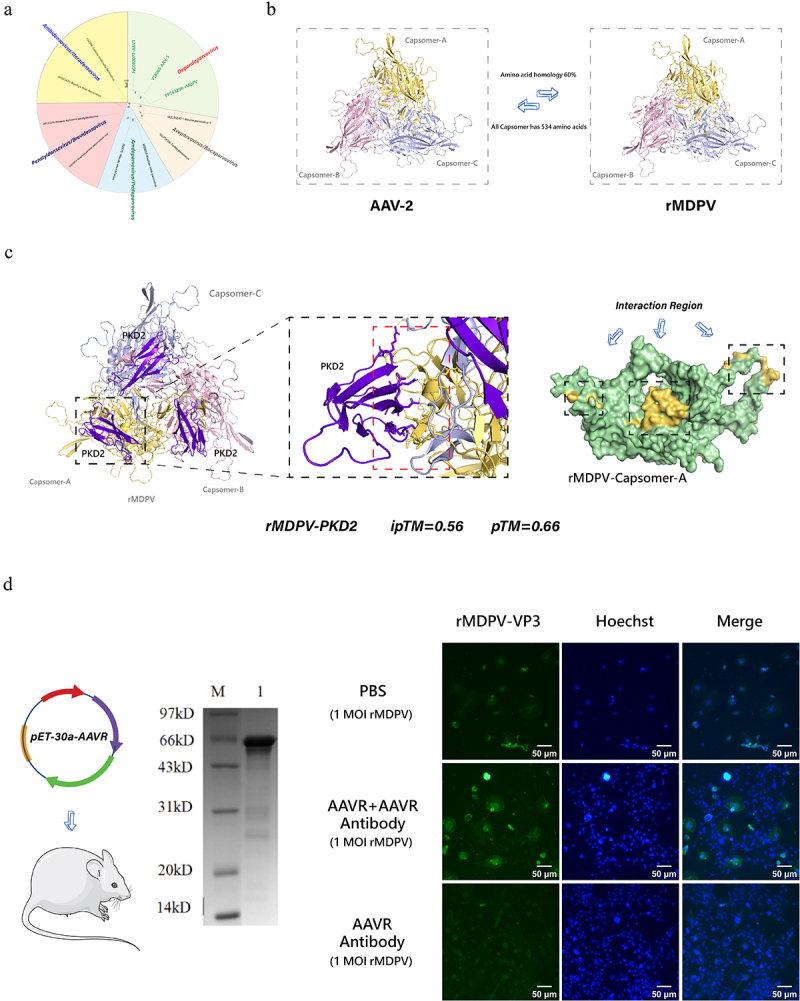
Protein – protein interaction network of WPVs. (a) Phylogenetic tree of *Parvoviridae*. (b) AAV2 and rMDPV capsid modelling. (c) Docking simulation and interaction region screening of rMDPV and PKD2 molecules, the interaction threshold was set as ipTM+pTM > 0.75 (d) Antibody blocking assay to verify AAVR as a potential receptor for rMDPV.

Based on the phylogenetic analysis of *Parvoviridae* (Figure 4a), rMDPV was classified within the genus *Dependoparvovirus*, along with AAV. Molecular modelling revealed a high structural similarity between rMDPV and AAV2 VP3 proteins, sharing 60% amino acid homology, with both exhibiting distinct canyon-like regions oriented towards the icosahedral five-fold axis of the viral particles (Figure 4b). Leveraging AAVR’s proteolytic cleavage properties, molecular docking simulations of pKD1-pKD5 with the rMDPV capsid unexpectedly identified pKD2 as exhibiting high binding affinity (ipTM+pTM scores > 0.75 threshold). PyMOL visualizations mapped several potential amino acid interaction sites at the docking interface. Except for a few sporadic amino acid residues, the main protein interactions were concentrated in three regions of the protein. We hypothesized that amino acid residue changes in these three regions could significantly affect the binding ability of WPVs to AAVR (Figure 4c). The antibody blocking assay results demonstrated distinct outcomes across experimental groups: (1) The negative control exhibited pronounced cytopathic effects (CPE) with progressively diminishing fluorescence signals due to cell death; (2) The positive control, where AAVR antibodies were pre-saturated with cognate protein, showed reduced cellular binding capacity, resulting in sporadic strong fluorescence signals in limited cells; (3) The test group displayed neither significant CPE nor intense fluorescence, presenting only weak viral signals, collectively indicating effective viral entry blockade by AAVR-specific antibodies (Figure 4d).

### Protein conformation analysis

**Figure 5.**
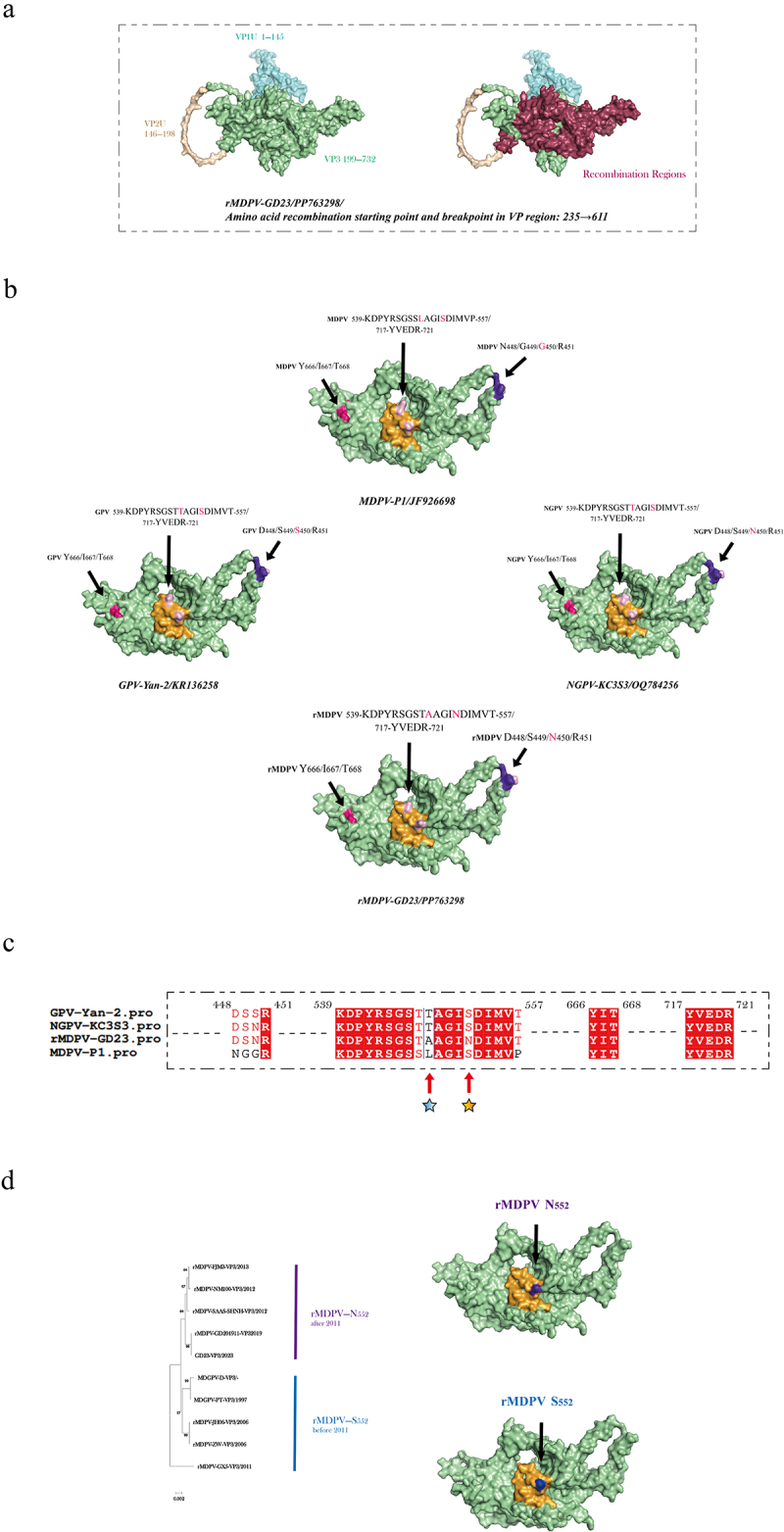
Protein modelling results for WPVs. (a) Modeling results of the VP protein of GD-23. (b) Modeling results of the VP3 protein of the standard WPV strain. (c) Homology comparison of potential interaction regions among different WPV strains. (d) Phylogenetic tree of rMDPV, along with the modelling results for the VP3 protein.

The complete capsid structure and recombination regions of rMDPV GD-23 are illustrated in Figure 5a. Notably, the VP3 region of GPV has replaced the majority of the VP3 region of MDPV (70.5% of the VP3 region was fully replaced). Notably, the model scoring results indicate that the VP1U and VP2U regions do not seem to be present in their natural state. Instead, the VP3 region alone forms the complete capsid structure of the virus in its natural environment. The VP1U and VP2U regions appear to be exposed and functional only during specific periods of viral infection due to changes in the conformational state of the viral proteins.

We selected standard strains representing four evolutionary directions of WPVs and performed structural modelling based on the VP3 amino acid sequence (Figure 5b). The terminal residues within the “canyon” region (highlighted in purple) are pivotal for determining the binding affinity between the AAV and AAVR. In classic MDPV, this region is characterized by the amino acid sequence “N-G-G-R,” whereas in classic GPV, it is represented by “D-S-S-R.” For NGPV and rMDPV, the sequence is “D-S-N-R,” with rMDPV exhibiting a substitution of serine with asparagine at this site compared to MDPV. Comparative analysis of WPVs at this position revealed that these four amino acids were highly conserved within each viral lineage. This conservation indicates that rMDPV, following recombination, may exhibit a distinct evolutionary pattern that could align with that of NGPV in certain aspects of its evolution.

The amino acid variation of WPV in the three interaction regions was compared and analysed, and it was found that there were obvious variations at two sites in rMDPV (Figure 5c). At amino acid position 548, rMDPV displays a unique variation not observed in other WPVs, and this variation is highly conserved among strains. In contrast, at position 552, the amino acid is not conserved across strains but shows a strong correlation with the temporal distribution of the strains. Phylogenetic analysis of rMDPV (Figure 5d) indicated that strains before 2011 differed from those after 2011 at this position, exhibiting a substitution of serine with asparagine. This variation facilitates the differentiation of evolutionary trajectories within rMDPV based on a specific amino acid position.

In summary, using the latest AlphaFold 3 for protein conformation modelling of rMDPV and drawing on the foundational research of AAV, we discovered that rMDPV undergoes a substitution of serine with asparagine at several potential key sites. This substitution prevents phosphorylation of the viral capsid at these sites, subsequently affecting the protein activity and conformational changes in the virus. This modification may be related to differences in pathogenicity and cross-species infections.

### Animal infection experiments

**Figure 6.**
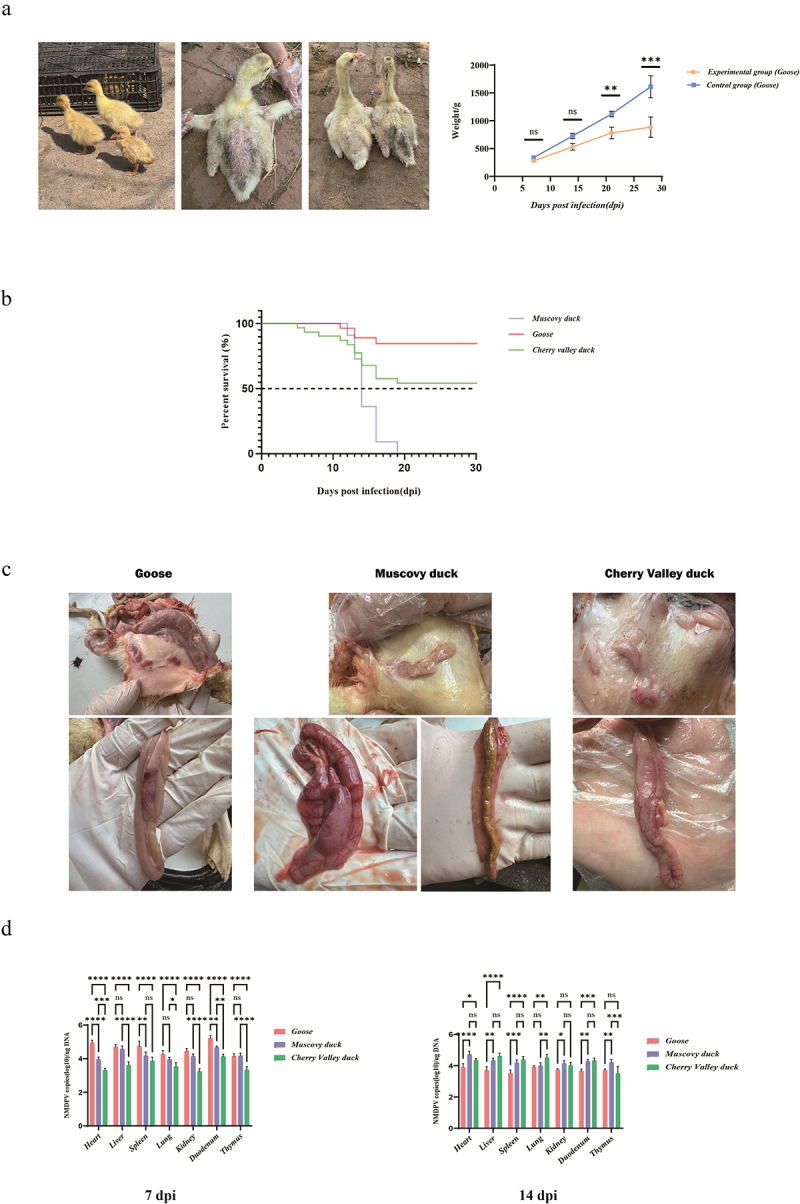
Infection of waterfowl with the rMDPV-GD-23 strain: changes in body weight, mortality rate, clinical characteristics, and viral load of infected birds. (a) Trends in clinical infection characteristics of the experimental group of geese and differences in body weight changes compared with the control group. (b) Survival rates of the different hosts. (c) Thymus and pancreas/duodenum lesion characteristics in different hosts. (d) Viral loads in the heart, liver, spleen, lungs, kidneys, duodenum, and thymus of different hosts. Data are expressed as mean ± SEM. All experiments were repeated at least thrice, and the results were similar. *p < 0.05, **p < 0.01, ***p < 0.001, **** *p* < 0.0001, indicating a statistically significant difference compared between the different groups.

The negative control group did not exhibit any characteristic signs of parvovirus infection throughout the study. Notably, geese displayed significant impairment in growth performance early in the infection, with pronounced feather loss on the back at approximately two weeks of age, a phenomenon not observed in the control group. Weight differences between healthy and experimental geese were recorded at four time points (7, 14, 21, and 28 dpi), revealing significant disparities beginning at 21 dpi (Figure 6a). In contrast, Cherry Valley and Muscovy ducks did not exhibit any typical pathological changes during the study period.

In terms of viral lethality, the rMDPV-GD-23 strain resulted in the mortality of all Muscovy ducks, and it also demonstrated a notably high mortality rate in Cherry Valley ducks, which reached 46.7% by the conclusion of the observation period. In contrast, the mortality rate in geese was only 13.3% (Figure 6b). Most fatalities among the experimental animals occurred approximately in the second week post-infection, coinciding with the period when the virus completes latent replication and begins to extensively disseminate viral particles to surrounding cells.

Following a meticulous examination of tissue samples from acute mortality cases, we performed a comparative analysis of the thymus and duodenum/pancreas, which exhibited characteristic parvoviral lesions (Figure 6c). The viral strain elicited marked thymic haemorrhage in geese, whereas the severity of infection in Muscovy and Cherry Valley ducks was comparatively lower. In the deceased Muscovy ducks, extensive haemorrhaging was observed in the duodenum and pancreas, whereas the geese demonstrated minimal infection. Conversely, pathological changes in Cherry Valley ducks were primarily characterized by intestinal swelling and irregular punctate haemorrhages within the pancreas. Fresh tissue samples (heart, liver, spleen, lungs, kidneys, duodenum, and thymus) were collected on days 7 and 14 to determine the viral load of rMDPV and compare the replication levels of the virus across the three types of hosts (Figure 6d). On the seventh day post-infection, the replication efficiency of the virus was highest in geese, with significant differences in viral load observed across various organs compared to that in Muscovy and Cherry Valley ducks. However, results at 14 days post-infection indicated a marked decline in viral load within geese, whereas a notable upward trend was observed in both Muscovy and Cherry Valley ducks, with significant differences in viral load between these species. This may be closely related to the virus’s capacity to effectively stimulate an immune response in geese.

## Discussion

RMDPV has recently emerged as a widespread and virulent pathogen in China, leading to significant mortality in Muscovy ducks and posing a considerable threat to the duck farming industry [21]. Following the isolation and whole-genome sequencing of the rMDPV-GD-23 strain, phylogenetic trees were constructed for various viral gene segments. Our analysis revealed that phylogenetic classification based on the VP3 gene is crucial for distinguishing between classic MDPV and recombinant strains within the broader evolutionary framework of WPVs. Furthermore, we identified two distinct evolutionary lineages within the recombinant strains, which could be delineated based on their temporal prevalence and specific amino acid variations. These findings suggest that rMDPV has undergone significant evolutionary changes in the recent past. Consistent with previous studies, recombination events in rMDPV predominantly occur in the P9 promoter and VP3 regions, which encompass 70% of the gene [12], indicating that the recombination pattern has remained stable throughout the virus’s evolution.

The genomes of wild-type strains exhibit exceptionally high rates of recombination and mutation during outbreaks, significantly affecting the identification and assessment of key viral sites. However, research on other viruses in the same family can provide valuable comparative insights [22]. Both AAV and WPVs belong to the *Parvoviridae family* [2]. However, owing to the considerable success of rAAV vectors, advancements in AAV research have considerably outpaced those of WPVs. In this study, based on the results of AAVR, we identified AAVR-PKD2 as a potential receptor for rMDPV from the perspective of a three-dimensional model competing with antibody receptors by screening AAVR and rMDPV interaction proteins. Following the interaction of amino acid site differences among WPVs, we identified two key amino acid mutations within the interacting region. These mutations involve the substitution of serine, which is highly susceptible to phosphorylation by eukaryotic cells, with asparagine, which is resistant to phosphorylation by eukaryotic cells. Such mutations in the Zika virus have been shown to significantly enhance viral infectivity in hosts [23]. We hypothesize that these sites hold significant research potential and plan to use reverse genetic tools for site-directed mutagenesis to further explore their biological significance.

MDPV has a long-standing prevalence in China. Recently, with the ongoing expansion of the aquaculture industry, recombinant strains of waterfowl parvoviruses have emerged, primarily as a result of recombination between GPV and MDPV [24]. Following complete adaptation to the host environment, these recombinant strains replicated the characteristic pathological manifestations of GPV, including diarrhoea and intestinal mucosal detachment, culminating in luminal obstruction in Muscovy ducks. Our experimental findings demonstrate that rMDPV exhibits adaptive capacity within Cherry Valley duck culture environments, with confirmed pathogenicity across multiple avian species, including geese, Muscovy ducks, and Cherry Valley ducks. The emergence of rMDPV provides compelling evidence of the frequent occurrence of cross-species recombination events among waterfowl parvoviruses. Such recombination mechanisms not only confer distinct cross-species infectivity profiles but also drive viral evolutionary divergence into a unique phylogenetic lineage within the GPV-related cluster.

Based on our results, we hypothesized that AAVR is a conserved interacting receptor of WPVs in waterfowl hosts, and that mutations in key amino acid sites on the capsid surface of WPVs may change the binding affinity of AAVR, leading to significant differences in infectivity among different hosts. For instance, the initial infection of Cherry Valley ducks with NGPV did not induce intestinal obstruction or high mortality rates in ducklings; instead, the virus had a notable inhibitory effect on host growth and development. This phenomenon may be attributed to the lower binding affinity of NGPV to Cherry Valley duck AAVR compared to the interaction between GPV and goose AAVR, leading to a distinct chronic infection profile in these ducks. In contrast, following extensive gene segment recombination, the further evolved rMDPV elicited pathological changes in Muscovy ducks akin to those seen in goslings, while exhibiting reduced pathogenicity in geese due to decreased AAVR affinity. The infection characteristics of recombinant duck parvovirus in geese are consistent with those of NGPV in Cherry Valley ducks, and infected geese exhibit developmental and feather growth disorders. Our animal trial results support the reliability of this hypothesis, and the GD-23 recombinant strain serves as a valuable model for studying viral evolution. Future research should focus on exploring additional key amino acid variants and developing reverse genetics tools for validation based on the genomic characteristics of this virus.

By integrating the AlphaFold 3 protein interaction prediction model with the AAVR research framework, this study provides the first evidence that members of the genus Parvovirus can achieve host recognition through shared AAVR receptors, offering novel insights into the mechanisms of viral-receptor evolution. Simultaneously, we predicted that two key amino acid mutations may mediate the cross-species transmission of rMDPV among Anseriformes by altering receptor-binding affinity. It should be noted that the precise molecular interaction network between AAVR and rMDPV remains incompletely resolved due to research resource constraints, although related mechanistic investigations are currently underway. Furthermore, the systematic identification of critical amino acid determinants governing viral cross-species transmission requires the implementation of deep mutational scanning and related technologies.

## Supplementary Material

Table_revision_R2 - Clean.docx

## Data Availability

The data that support the findings of this study are openly available in figshare at https://doi.org/10.6084/m9.figshare.27230178.v6.

## References

[1] Emmanuel SN, Mietzsch M, Tseng YS, et al. Parvovirus capsid-antibody complex structures reveal conservation of antigenic epitopes across the family. Viral Immunol. 2021;34(1):3–14. doi: 10.1089/vim.2020.0022 32315582 PMC8020512

[2] Kailasan S, Agbandje-McKenna M, Parrish CR. Parvovirus family conundrum: what makes a killer? Annu Rev Virol. 2015;2(1):425–450. doi: 10.1146/annurev-virology-100114-055150 26958923

[3] Cotmore SF, Agbandje-McKenna M, Chiorini JA, et al. The family *parvoviridae*. Arch Virol. 2014;159(5):1239–1247. doi: 10.1007/s00705-013-1914-1 24212889 PMC4013247

[4] Glavits R, Zolnai A, Szabo E, et al. Comparative pathological studies on domestic geese (Anser anser domestica) and Muscovy ducks (Cairina moschata) experimentally infected with parvovirus strains of goose and Muscovy duck origin. Acta Vet Hung. 2005;53(1):73–89. doi: 10.1556/AVet.53.2005.1.8 15782661

[5] Zadori Z, Stefancsik R, Rauch T, et al. Analysis of the complete nucleotide sequences of goose and muscovy duck parvoviruses indicates common ancestral origin with adeno-associated virus 2. Virology. 1995;212(2):562–573. doi: 10.1006/viro.1995.1514 7571426

[6] Wang J, Duan J, Meng X, et al. Cloning of the genome of a goose parvovirus vaccine strain SYG61v and rescue of infectious virions from recombinant plasmid in embryonated goose eggs. J Virol Methods. 2014;200:41–46. doi: 10.1016/j.jviromet.2014.02.014 24565999

[7] Le gall-Recule G, Jestin V. Biochemical and genomic characterization of muscovy duck parvovirus. Arch Virol. 1994;139(1–2):121–131. doi: 10.1007/BF01309459 7826205

[8] Tatar-Kis T, Mato T, Markos B, et al. Phylogenetic analysis of Hungarian goose parvovirus isolates and vaccine strains. Avian Pathol. 2004;33(4):438–444. doi: 10.1080/03079450410001724067 15370042

[9] Shen H, Huang J, Yan Z, et al. Isolation and characterization of a recombinant Muscovy duck parvovirus circulating in Muscovy ducks in South China. Arch Virol. 2020;165(12):2931–2936. doi: 10.1007/s00705-020-04829-7 33011831

[10] Huo X, Chen Y, Zhu J, et al. EvolutiEvolutiOn, genetic recombination, and phylogeography of goose parvovirus. Comp Immunol Microbiol Infect Dis. 2023;102:102079. doi: 10.1016/j.cimid.2023.102079 37812834

[11] Wang S, Cheng XX, Chen SY, et al. Genetic characterization of a potentially novel goose parvovirus circulating in Muscovy duck flocks in Fujian Province, China. J Vet Med Sci. 2013;75(8):1127–1130. doi: 10.1292/jvms.12-0527 23563621

[12] Wang J, Mi Q, Wang Z, et al. Sole recombinant Muscovy duck parvovirus infection in Muscovy ducklings can form characteristic intestinal embolism. Vet Microbiol. 2020;242:108590. doi: 10.1016/j.vetmic.2020.108590 32122594

[13] Zhu Y, Zhou Z, Huang Y, et al. Identification of a recombinant Muscovy duck parvovirus (MDPV) in Shanghai, China. Vet Microbiol. 2014;174(3–4):560–564. doi: 10.1016/j.vetmic.2014.10.032 25465183

[14] Zhang R, Xu G, Cao L, et al. Divergent engagements between adeno-associated viruses with their cellular receptor AAVR. Nat Commun. 2019;10(1):3760. doi: 10.1038/s41467-019-11668-x 31434885 PMC6704107

[15] Smith DH, Ward P, Linden RM. Comparative characterization of rep proteins from the helper-dependent adeno-associated virus type 2 and the autonomous goose parvovirus. J Virol. 1999;73(4):2930–2937. doi: 10.1128/JVI.73.4.2930-2937.1999 10074142 PMC104052

[16] Thakur AK, Fezio WL. A computer program for estimating LD50 and its confidence limits using modified Behrens-Reed-Muench cumulant method. Drug Chem Toxicol. 1981;4(3):297–305. doi: 10.3109/01480548109018136 7338208

[17] Nguyen LT, Schmidt HA, von Haeseler A, et al. IQ-TREE: a fast and effective stochastic algorithm for estimating maximum-likelihood phylogenies. Mol Biol Evol. 2015;32(1):268–274. doi: 10.1093/molbev/msu300 25371430 PMC4271533

[18] Abramson J, Adler J, Dunger J, et al. Accurate structure prediction of biomolecular interactions with AlphaFold 3. Nature. 2024;630(8016):493–500. doi: 10.1038/s41586-024-07487-w 38718835 PMC11168924

[19] Rosignoli S, Paiardini A. Boosting the full potential of PyMOL with structural biology plugins. Biomolecules. 2022;12(12):1764. doi: 10.3390/biom12121764 36551192 PMC9775141

[20] Homma F, Huang J, van der Hoorn RAL. AlphaFold-Multimer predicts cross-kingdom interactions at the plant-pathogen interface. Nat Commun. 2023;14(1):6040. doi: 10.1038/s41467-023-41721-9 37758696 PMC10533508

[21] He J, Zhang Y, Hu Z, et al. Recombinant Muscovy duck parvovirus led to ileac damage in Muscovy ducklings. Viruses. 2022;14(7):1471. doi: 10.3390/v14071471 35891451 PMC9315717

[22] Alsafi RT. Lessons from SARS-CoV, MERS-CoV, and SARS-CoV-2 infections: what we know so far. Can J Infect Dis Med Microbiol. 2022;2022:1156273. doi: 10.1155/2022/1156273 35992513 PMC9391183

[23] Yuan L, Huang XY, Liu ZY, et al. A single mutation in the prM protein of Zika virus contributes to fetal microcephaly. Science. 2017;358(6365):933–936. doi: 10.1126/science.aam7120 28971967

[24] Shen H, Zhang W, Wang H, et al. Identification of recombination between Muscovy duck parvovirus and goose parvovirus structural protein genes. Arch Virol. 2015;160(10):2617–2621. doi: 10.1007/s00705-015-2541-9 26239342

