# Supplementary material for: The study on the molecular characteristics and variation patterns of the recombinant Muscovy duck parvovirus strain GD-23
Source: Virulence. 2025 Jul 15;16(1):2530666. doi: 10.1080/21505594.2025.2530666 (PMC12269699; doi:10.1080/21505594.2025.2530666)
Supplement: Table_revision_R2 - Clean.docx [file KVIR_A_2530666_SM2282.docx]

**The study on the molecular characteristics and variation patterns of the recombinant Muscovy duck parvovirus strain GD-23.**

Mingtian Mao^1,2,3^, Jiake Li^1,2,3^, Caiqi Wang^1,2,3^, Mian Wu^1,2,3^, ChengGuang Lu^1,2,3^, Yudong Zhu^1,2,3^, HuiHui Li^4^, Bing Li^4^, Meixi Lu^4^

Yi Tang^4*^

1. College of Animal Science and Technology, Shandong Agricultural University, 61 Daizong Street, Tai’an, Shandong Province, 271018, China

2. Shandong Provincial Key Laboratory of Animal Biotechnology and Disease Control and Prevention, Tai’an, Shandong, 271018, China

3. Shandong Provincial Engineering Technology Research Center of Animal Disease Control and Prevention, Tai’an, Shandong, 271018, China

4. Institute of Animal Science, Chinese Academy of Agricultural Sciences, Beijing 100193, China

*Corresponding authors: Yi Tang ([tyck288@163.com](mailto:tyck288@163.com))

**Table S1.** Primers used for PCR amplification in this study

| Name | Sequence of primers(5′to3′) | Length |
| --- | --- | --- |
| GD23-1F | CTCATTGGAGGGTTCGTTCGTTCGAAC | 194 bp |
| GD23-1R | GCATGCGCCCGATCAGCCTTGACAAC |  |
| GD23-2F | GCATGCGCCCGATCTGCCATGAAAATT | 2166 bp |
| GD23-2R | TCATTCATGTCATCTCTTAGTTCATTA |  |
| GD23-3F | AGAGTCTGGGTGAAAACTTTTGTAAT | 1856 bp |
| GD23-3R | CCACCCTACACCGTTTGTAGGTGCTAC |  |
| GD23-4F | CACTGCAGCAGGAATAAATGATATTA | 883 bp |
| GD23-4R | GCATGCGCCCGATCAGCCTTGACAACC |  |
| GD23-5F | TTTCCGGTTGTCAAGGCTGATCGGGCG | 225 bp |
| GD23-5R | CTCATTGGAGGGTTCGTTCGTTCGAA |  |

**Table S2.** GenBank accession numbers of Waterfowl parvovirus for sequence alignment

| *Parvoviridae* | Virus strain | Origin | Year of isolation | Host | GenBank accession  (complete genome) |
| --- | --- | --- | --- | --- | --- |
| GPV | Y | China | 2014 | Muscovy duck | KC178571 |
| GPV | Yan-2 | China | 2015 | Yan goose | KR136258 |
| GPV | YZ99-6 | China | 2015 | Goose | KC996730 |
| GPV | LH | China | 2014 | Goose | KM272560 |
| GPV | SH | China | 2016 | Anser anser | JF333590 |
| GPV | B | Hungary | 1995 | Anser anser | U25749 |
| GPV | SYG61-v | China | 2015 | Goose | KC996729 |
| GPV | Vaccine-VG32/1 | German | unknown | Goose | EU583392 |
| GPV | SHFX1201 | China | 2013 | Swan | KC478066 |
| GPV | 82-0321 | Chian | 2004 | Goose | AY382883 |
| GPV | 82-0321v | China | 2008 | Goose | EU583389 |
| N-GPV | GXN45 | China | 2020 | Cherry Valley duck | MH717783 |
| N-GPV | AH | China | 2019 | Cherry Valley duck | MH444513 |
| N-GPV | HN1P | China | 2019 | Anas platyrhynchos | MK737642 |
| N-GPV | JS1123 | China | 2020 | Cherry Valley duck | MN415967 |
| N-GPV | HuN18 | China | 2019 | Linwu Sheldrake | MK736656 |
| N-GPV | JS1212 | China | 2020 | Anas platyrhynchos | MT084127 |
| N-GPV | SD0218 | China | 2020 | Cherry Valley duck | MN415968 |
| N-GPV | SDDY1605 | China | 2017 | Cherry Valley ducks | MF441224 |
| N-GPV | SDHZ1604 | China | 2017 | Cherry Valley ducks | MF441223 |
| N-GPV | JS1603 | China | 2017 | Cherry Valley ducks | MF441226 |
| N-GPV | AH1606 | China | 2017 | Cherry Valley ducks | MF441225 |
| N-GPV | SDLY1512 | China | 2017 | Cherry Valley ducks | MF441221 |
| MDPV | P1 | China | 2011 | Muscovy duck | JF926698 |
| MDPV | FM | France | 1997 | Cairina moschata | NC006147 |
| MDGPV | D | China | 2011 | muscovy duckling | JF926696 |
| MDGPV | GX5 | China | 2015 | Muscovy duck | KM093740 |
| rMDPV | PT | China | 1997 | Cairina moschata | NC006147 |
| rMDPV | ZW | China | 2017 | Muscovy duck | KY744743 |
| rMDPV | JH06 | China | 2019 | Muscovy duck | MH807697 |
| rMDPV | FJM3 | China | 2018 | Muscovy duck | KR075690 |
| rMDPV | NM100 | China | 2018 | Cairina moschata | KU641556 |
| rMDPV | GD201911 | China | 2021 | Cairna moschata | MT450871 |
| rMDPV | GD23 | China | 2024 | Cairina moschata | PP763298 |
| rMDPV | SAAS-SHNH | China | 2013 | Muscovy duck | KC171936 |
| AAV | AAV1 | USA | 2018 | / | NC002077 |
| AAV | AAV5 | FRG | 1999 | / | Y18065 |
| AAV | AAV2 | USA | 2025 | / | PD149030 |
| BPV | 1 | USA | 2007 | Bovine | DQ335247 |
| TPV | 260 | USA | 2010 | Turkey | GU214706 |
| ADV | G | USA | 1995 | Aleutian mink | M20036 |
| MVM | P | USA | 1995 | Mice | J02275 |
| AgDNV | / | USA | 2008 | Anopheles gambiae | EU233812 |
| IHHNV | 1 | USA | 2014 | Shrimp | AF273215 |
| BmBDV | 1 | Canada | 2002 | Bombyx mori | AY033435 |
| GmDNV | 1 | USA | 2003 | Galleria mellonella | L32896 |

**Table S3.** The assessment results of the Ramachandran plot for the four model categories

| Ramachandran plot  residues in (%) | GPV/Yan-2/VP3  KR136258 | MDPV/P1/VP3  JF926698 | NGPV/KC3S3/VP3  OQ784256 | rMDPV/GD23/VP3  PP763298 |
| --- | --- | --- | --- | --- |
| Most favoured regions | 92.6 | 92.4 | 93.0 | 93.0 |
| Additional allowed regions | 6.5 | 6.6 | 6.1 | 6.3 |
| Generously allowed regions | 0.9 | 0.9 | 0.9 | 0.7 |
| Disallowed regions | 0 | 0 | 0 | 0 |

| Ramachandran plot  residues in (%) | AAV2  PD149030 | rMDPV/GD23  PP763298 | AAVR-PKD2  KIAA0319L |
| --- | --- | --- | --- |
| Most favoured regions | 94.6 | 93.1 | 93.2 |
| Additional allowed regions | 4.5 | 6.0 | 6.1 |
| Generously allowed regions | 0.9 | 0.9 | 0.7 |
| Disallowed regions | 0 | 0 | 0 |
